# Supplementary material for: Arf GTPases Are Required for the Establishment of the Pre-Assembly Compartment in the Early Phase of Cytomegalovirus Infection
Source: Life (Basel). 2021 Aug 23;11(8):867. doi: 10.3390/life11080867 (PMC8399710; doi:10.3390/life11080867)
Supplement: Supplementary file 1 [file life-11-00867-s001.zip › life-1336729-supplementary.pdf]

life

Supplementary Materials

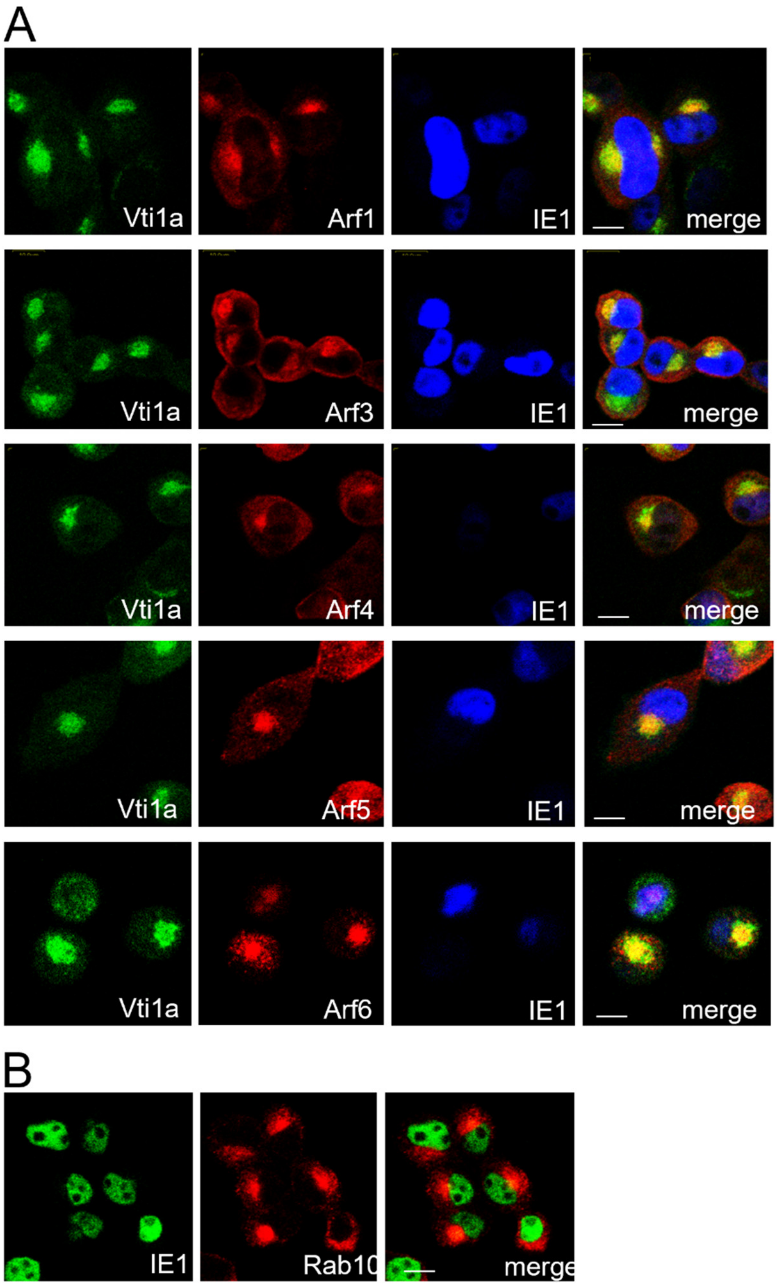

Figure S1. Colocalization of Arf proteins with Vti1a, inner pre-AC marker, and pre-AC development in untransfected cells. (**A**) Δm138-MCMV infected Balb 3T3 cells were fixed at 16 hpi, permeabilized, and stained against Arf protein (red fluorescence), Vti1a (green fluorescence), and IE1 (blue fluorescence). (**B**) Untransfected Balb 3T3 cells were infected with Δm138-MCMV, and at 16 hpi, cells were fixed, permeabilized, and stained against Rab10 (red fluorescence) and IE1 (green fluorescence). Focal-plane images across the mid-section of the cells are shown. Bars, 10 μm.

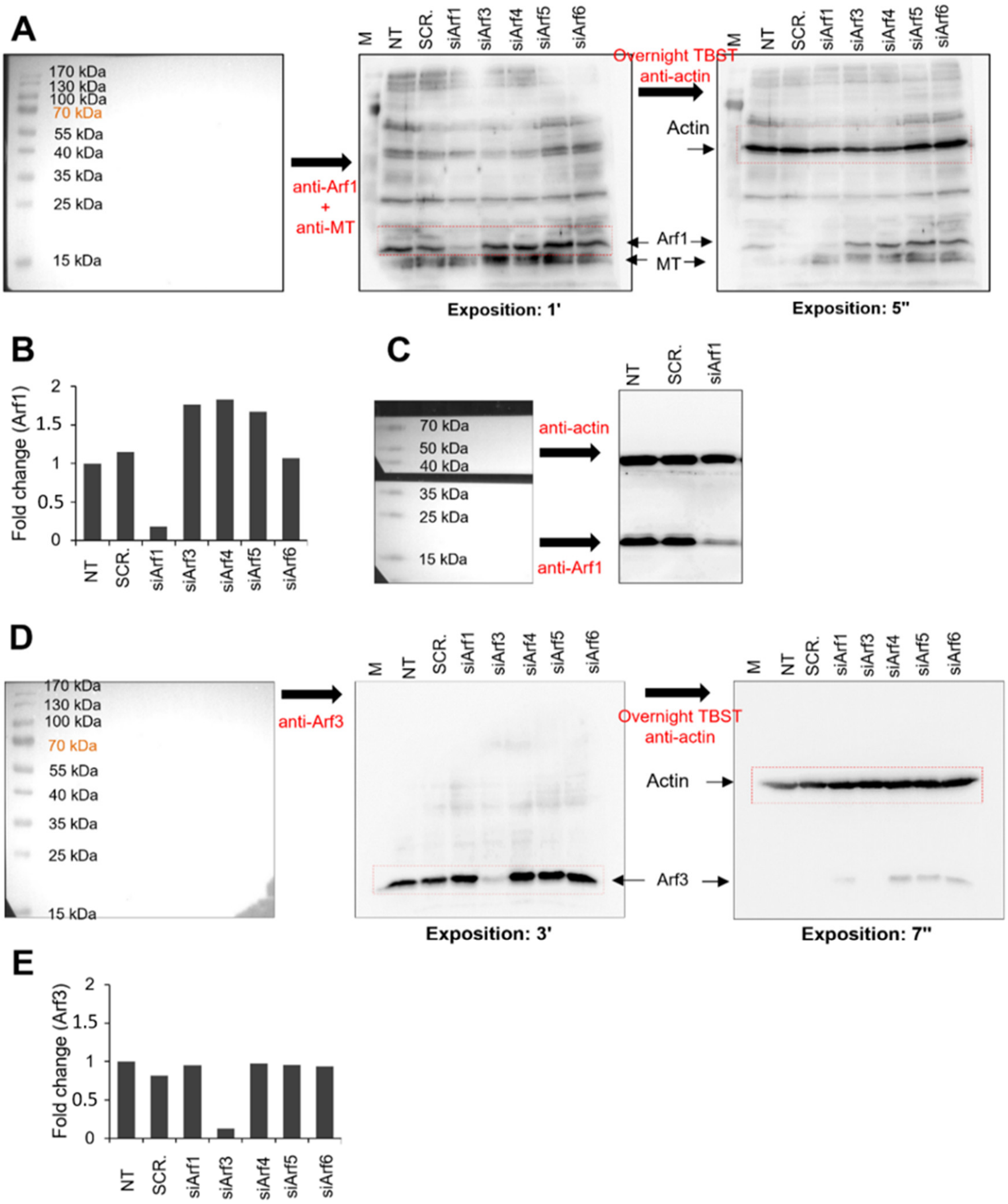

Figure S2. Original raw blots and unedited ECL images used as a representative Western blot in Figure 7 of the manuscript**.** Balb 3T3 cells were transfected with negative control scrambled siRNA (SCR.) or siRNAs targeting Arf proteins (siArf1-6). After 72 hours, expression of Arf1 and Metallothionein (**A**) or Arf3 (**D**) in untransfected and transfected cells was analyzed by Western blot. Actin was used as a loading control**.** Areas marked with a red dashed line are shown in Figure 7. (**C**) The expression of Arf1 in untransfected and transfected cells was analyzed by Western blot to show the specificity of the anti-Arf1 antibodies and to confirm that additional bands in (**A**) are the consequence of anti-MT staining. Actin was used as a loading control**.** Quantitative analysis of Arf1 (**B**) and Arf3 (**E**) expression levels in NT, SCR., and siArf1-6 transfected cells was performed as described in *Materials and methods.* Results are expressed as a fold change relative to Arf1 and Arf3 expression in NT cells.

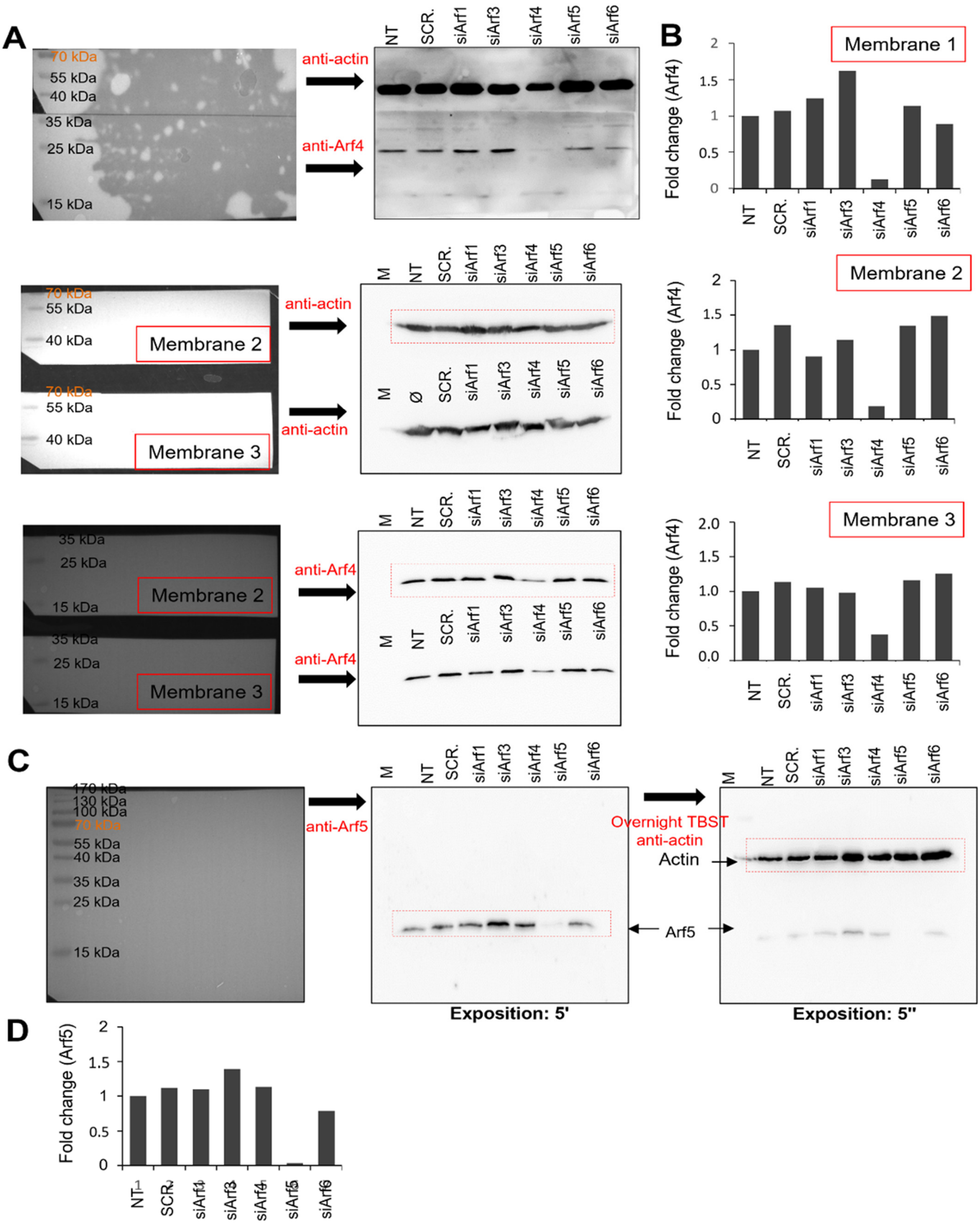

Figure S3. Original raw blots and unedited ECL images used as a representative Western blot in Figure 7 of the manuscript. Balb 3T3 cells were transfected with negative control scrambled siRNA (SCR.) or siRNAs targeting Arf proteins (siArf1-6). After 72 hours, expression of Arf4 (**A**) and Arf5 (**C**) in untransfected and transfected cells was analyzed by

Western blot. Actin was used as a loading control. Areas marked with a red dashed line are shown in Figure 7. Quantitative analysis of Arf4 (**B**) and Arf5 (**D**) expression levels in NT, SCR., and siArf1-6 transfected cells was performed as described in *Materials and methods.* Results are expressed as a fold change relative to Arf4 and Arf5 expression in NT cells.

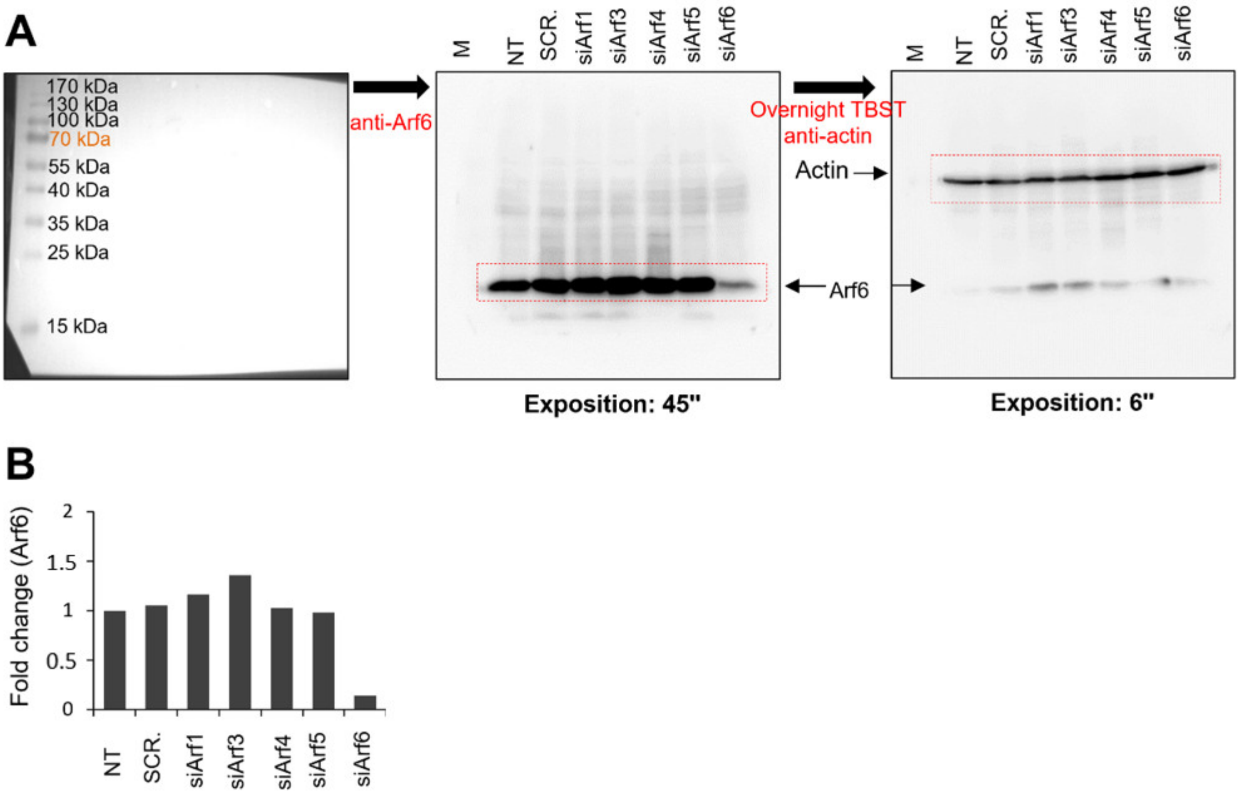

Figure S4. Original raw blots and unedited ECL images used as a representative Western blot in Figure 7 of the manuscript. **(A)** Balb 3T3 cells were transfected with negative control scrambled siRNA (SCR.) or siRNAs targeting Arf proteins (siArf1-6). After 72 hours, expression of Arf6 in untransfected and transfected cells was analyzed by Western blot. Actin was used as a loading control. Areas marked with a red dashed line are shown in Figure 7. (**B**) Quantitative analysis of Arf6 expression levels in NT, SCR., and siArf1-6 transfected cells was performed as described in *Materials and methods.* Results are expressed as a fold change relative to Arf6 expression in NT cells.

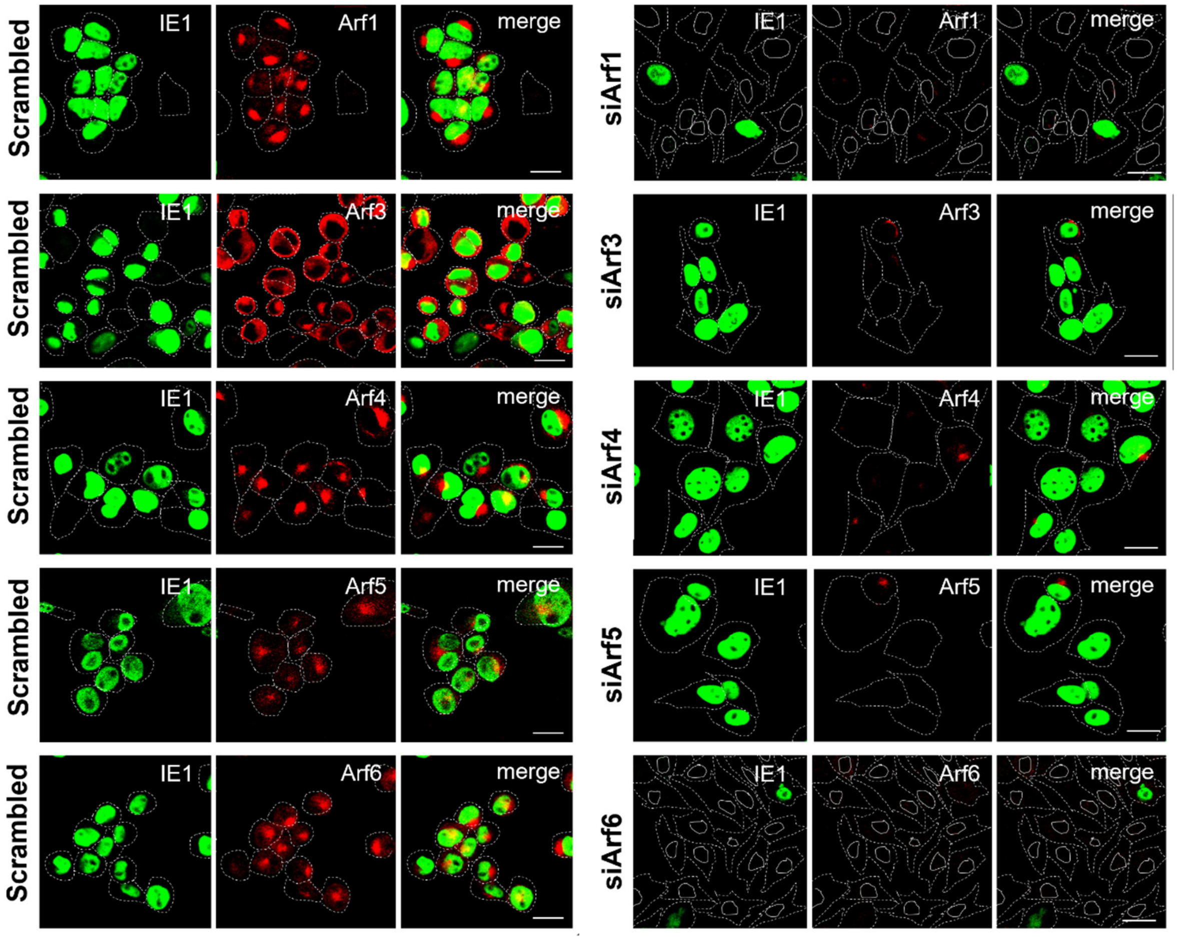

Figure S5. The efficiency of Arf1, Arf3, Arf4, Arf5, and Arf6 silencing in immunofluorescence experiments**.** Balb 3T3 cells were transfected with negative control scrambled siRNA (SCR.) or siRNAs targeted Arf1-6 (siArf1-6) and after 72 hrs infected with Δm138-MCMV. At 16 hpi, cells were fixed, permeabilized, and stained against indicated Arf protein (red fluorescence) and IE1 (green fluorescence). Cell borders are indicated by dashed and nuclei by full lines. Representative images with focal planes across the mid-section of the cells are shown. Bars, 20 μm.

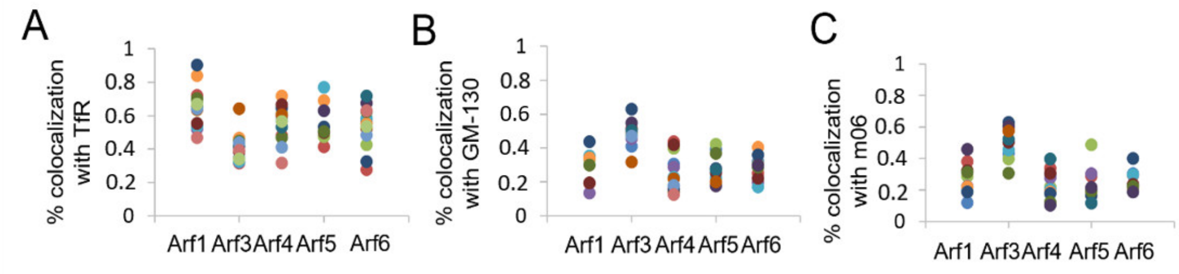

Figure S6. Individual data used for calculation of mean ± STDEV in colocalization analysis. Images were analyzed through the entire z-stacks for colocalization of Arf proteins and internalized TfR (**A**) or GM130 (**B**) or early viral protein m06 (**C**) by using Mander's coefficients of pixel overlap. Menders coefficients for each cell analyzed are presented. Data were used for the calculation of mean ± STDEV values that are given in Figures 4 (**A**), 5 (**B**), and 6 (**C**).

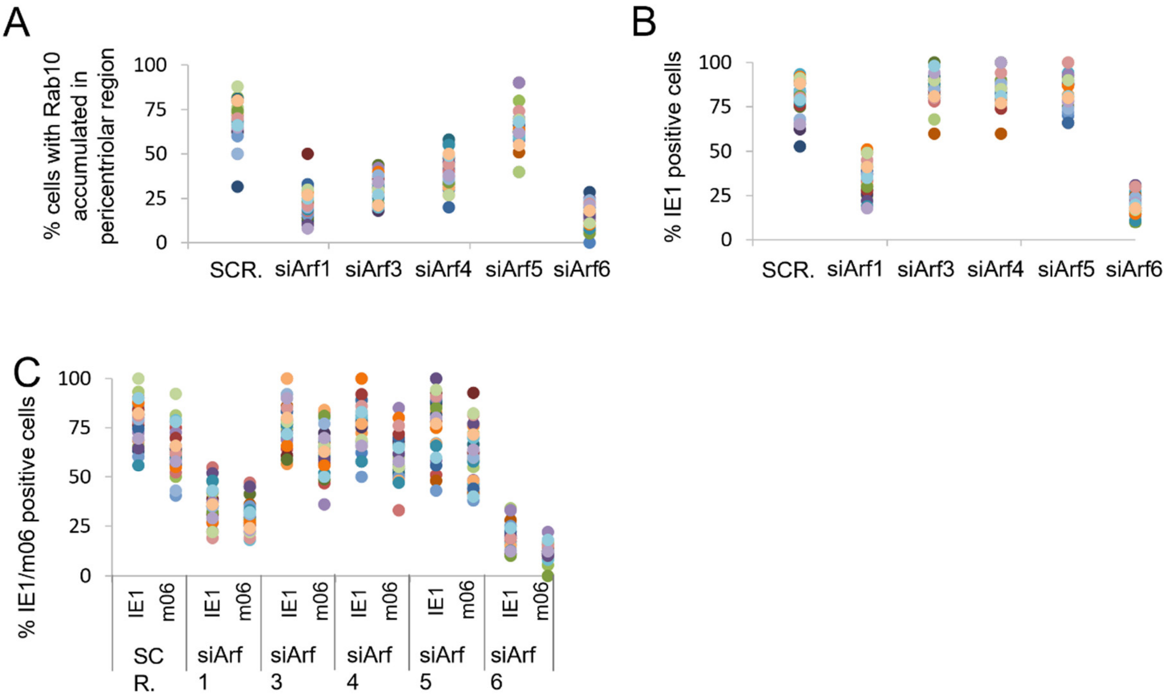

Figure S7. Individual data used for calculation of the mean ± STDEV in immunofluorescence knock-down experiments. Balb 3T3 cells were transfected with negative control scrambled siRNA (SCR.) or siRNAs targeted Arf1-6 (siArf1-6) and after 72 hrs infected with Δm138-MCMV. At 0 and 16 hpi, cells were fixed, permeabilized, and stained against IE1 and Rab10 or m06. Percentages of cells with the juxta-nuclear Rab10 accumulation **(A),** nuclear IE1 expressions (**B and C**) as well as cytoplasmatic m06 expression **(C)** were determined as described in Materials and Methods. The data are presented as percentage of positive cells per field of view/image. Data were used for the calculation of mean ± STDEV values that are given in Figures 8 (**A, B**) and 9 (**C**).

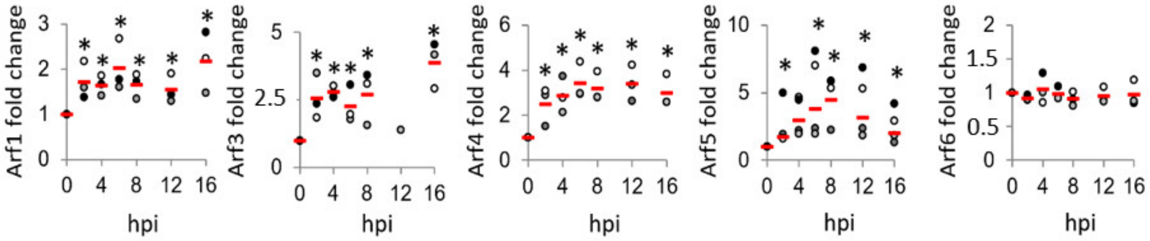

Figure S8. Quantitative analysis of expression levels of Arf proteins from at least three independent experiments during the early phase of MCMV infection. Balb 3T3 cells were infected with Δm138-MCMV, and the expression level of Arf proteins in the early phase of MCMV infection (0-16 hpi) was determined by Western blot. Quantitative analysis of their expression levels was performed as described in *Materials and methods.* Results represent quantification of WBs shown in Figures 1A, 1D, 2A, 2D, and 3A as well as two other independent WBs for each Arf that was analysed. Results are expressed as a fold change relative to Arf expression in 0 hpi MCMV infected cells. Circles represent individual experiments and red lines represent mean values. Asterisks indicate statistical significance ($p < 0.05$) when compared with 0 hpi.

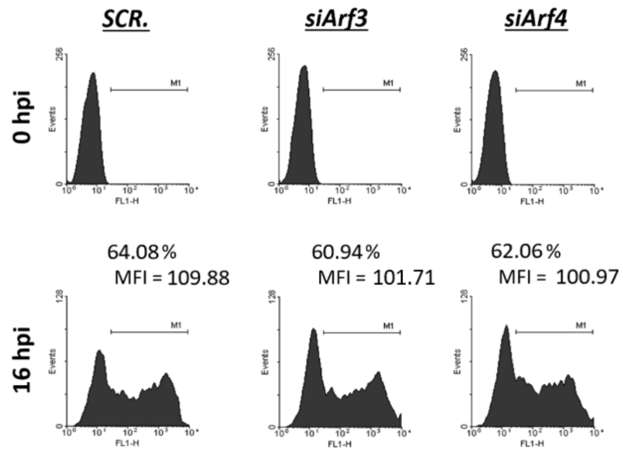

Figure S9. Effect of Arf3 and Arf4 knockdowns on IE1-GFP expression. Balb 3T3 cells transfected with scrambled siRNA (SCR.), siArf3, or siArf4 were 72 hours after transfection infected with C3X-GFP MCMV and at 0 and 16 hpi GFP fluorescent signal was analysed by flow cytometry.

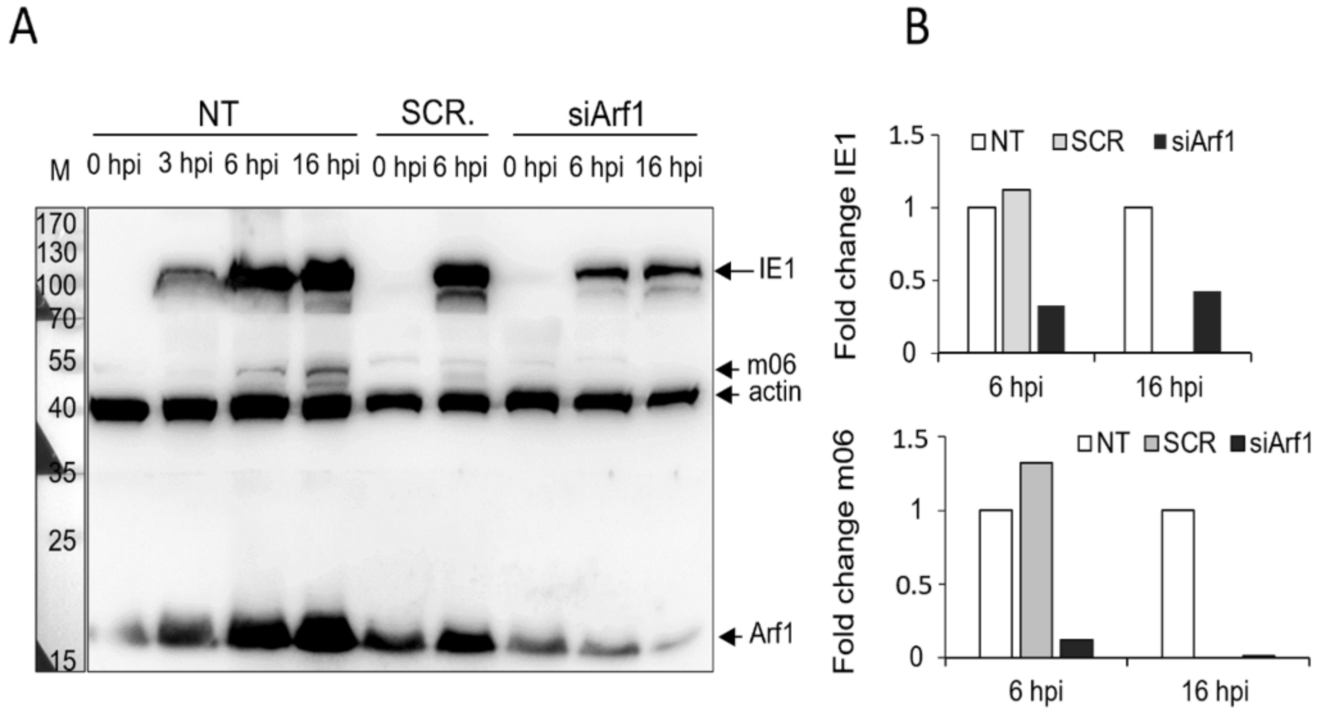

Figure S10. Knock-down of Arf1 inhibits expression of IE1 and m06 viral proteins. Untransfected (NT) Balb 3T3 cells or transfected with scrambled siRNA (SCR.) or siArf1 were 72 hrs after transfection infected with Δm138-MCMV(**B**), and at indicated time points post infection, the expression of IE1 and m06 was analysed by Western blot(**A**). Actin was used as a loading control and Arf1 expression was used as a control of silencing efficiency.
